# Cellular Response to Individual Components of the Platelet Concentrate

**DOI:** 10.3390/ijms22094539

**Published:** 2021-04-26

**Authors:** Vera Sovkova, Karolina Vocetkova, Věra Hedvičáková, Veronika Hefka Blahnová, Matěj Buzgo, Evzen Amler, Eva Filová

**Affiliations:** 1 Department of Biophysics, 2nd Faculty of Medicine, Charles University, V Uvalu 84, 150 06 Prague, Czech Republic; veronika.blahnova@iem.cas.cz (V.H.B.); evzen.amler@lfmotol.cuni.cz (E.A.); 2 Department of Tissue Engineering, Institute of Experimental Medicine, The Czech Academy of Sciences, Videnska 1083, 142 20 Prague, Czech Republic; karolina.vocetkova@iem.cas.cz (K.V.); vera.hedvicakova@iem.cas.cz (V.H.); eva.filova@iem.cas.cz (E.F.); 3 University Centre for Energy Efficient Buildings, Czech Technical University in Prague, Trinecka 1024, 273 43 Bustehrad, Czech Republic; matej@inocure.cz

**Keywords:** platelets, plasma, mesenchymal stem cells, fibroblasts

## Abstract

Platelet concentrates and especially their further product platelet lysate, are widely used as a replacement for cell culturing. Platelets contain a broad spectrum of growth factors and bioactive molecules that affect cellular fate. However, the cellular response to individual components of the human platelet concentrate is still unclear. The aim of this study was to observe cellular behavior according to the individual components of platelet concentrates. The bioactive molecule content was determined. The cells were supplemented with a medium containing 8% (*v*/*v*) of platelet proteins in plasma, pure platelet proteins in deionized water, and pure plasma. The results showed a higher concentration of fibrinogen, albumin, insulin growth factor I (IGF-1), keratinocyte growth factor (KGF), and hepatocyte growth factor (HGF), in the groups containing plasma. On the other hand, chemokine RANTES and platelet-derived growth factor bb (PDGF-bb), were higher in the groups containing platelet proteins. The groups containing both plasma and plasma proteins showed the most pronounced proliferation and viability of mesenchymal stem cells and fibroblasts. The platelet proteins alone were not sufficient to provide optimal cell growth and viability. A synergic effect of platelet proteins and plasma was observed. The data indicated the importance of plasma in platelet lysate for cell growth.

## 1. Introduction

Platelets are small, anucleated cellular fragments, widely used in many types of products as a source of growth factors involved in wound healing. Their administration for wound healing improvement is highlighted in fields such as sports medicine [1,2,3,4], dermatology [5,6,7,8,9,10,11,12,13] and dentistry [14,15,16,17,18]. Once the platelets activate, the growth factors are released and involved throughout the wound healing cascade. Growth factors play an important role in intracellular communication and in the control of cellular fate [19]. They regulate proteosynthesis, differentiation, migration and proliferation of target cells, due to the formation of complexes with membrane receptors. Each growth factor has one or more functions depending on the type of target cells and the specific conditions of the surrounding microenvironment [20].

There are several platelet products available, differing according to the original source of platelets, preparation procedure, representation of individual components, and target use [21].

Platelet lysate is a product based on the disruption of platelet membrane and release of the internal content into the solution. The individual components of platelet lysate and their ratio significantly influence the resulting effect of wound healing; however the contribution of individual components to the overall state has so far not been extensively monitored.

Generally, plasma, platelet bioactive molecules and additive solution are usually present in the platelet lysate. Depending on the preparation procedure, the ratio and presence of the individual components may vary among the resulting products. The effect of platelet bioactive molecules on wound healing has been monitored and described in many articles [8,11,22,23,24,25,26]. The effect of plasma, another component of platelet lysate, is also important. Plasma contains growth factors and immunoglobulins which are absent in platelets and can significantly increase the effect of products containing platelets. Immunoglobulins can serve as carrier molecules for some cytokines and growth factors, and enhance their activity [27]. Growth factors such as IGF-I, the most important growth factor involved in wound healing, is only found in plasma [28]. For this reason, it is advantageous to preserve the plasma in platelet lysate to the maximum level possible in autologous administration. Its beneficial effect was also observed in the allogeneic topical use of platelet lysate; therefore, the advantages of the presence of plasma outweigh the possible immunogenicity of the product [29,30]. Moreover, there is a clear beneficial effect of plasma as a component of platelet lysate for cell culture purposes.

The aim of this study was to compare and resolve the influence of human plasma and/or platelet lysate produced by different methods. Three types of blood derivatives were prepared in human platelet lysate (hPL), human platelet lysate in deionized water (hPL dH_2_O group) and human platelet poor plasma (hPPP group), and characterized for cytokines and growth factor panel. Murine fibroblasts and human mesenchymal stem cells (MSCs) were cultured, and the cell proliferation and viability were tested. We found the synergistic effect of plasma and platelet bioactive molecules to ensure the cells growth and viability.

## 2. Results

### 2.1. The Concentration of Overall Protein and Bioactive Molecules

The three compounds of platelet concentrate were prepared. Firstly, the hPL group contained platelet proteins in plasma and an additive solution, the hPL dH_2_O group contained platelet proteins in deionized water, and the hPPP group consisted of plasma and an additive solution without platelet proteins.

**Figure 1:**
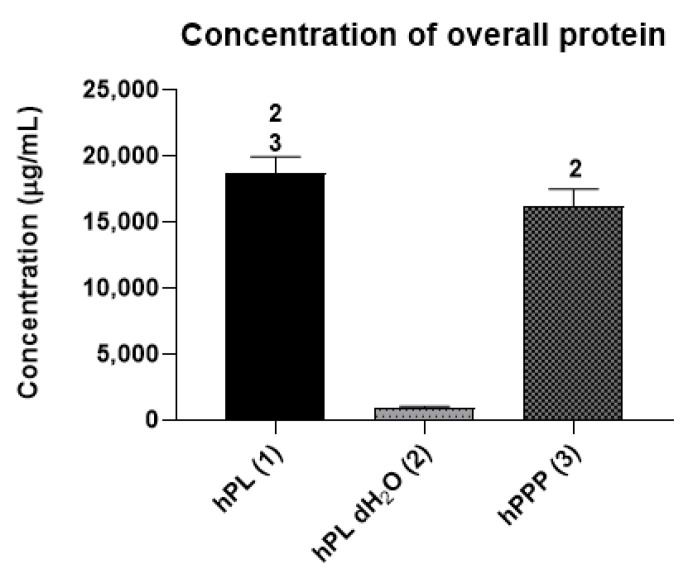
The overall protein concentration in the tested group (human platelet lysate—hPL group; human platelet lysate in deionized water—hPL dH_2_O group; platelet poor plasma—hPPP group; statistical analysis *p* < 0.05). The level of significance is denoted by the numbers above the bars in the graph. The numbers above the bars denote the type of the respective sample 1—hPL, 2—hPL dH_2_O, 3—hPPP. The significance was only denoted with a number above the bars with the significantly higher values.

The concentration of the overall protein (Figure 1) was significantly higher in the groups with plasma (hPL, hPPP) in comparison to the group containing only platelets (hPL dH_2_O).

The concentrations of fibrinogen, albumin and IGF-1 were evaluated (Table 1). All observed markers were under the detection limit in the group containing the platelets only (hPL dH_2_O). The remaining groups showed almost identical concentrations of selected markers.

**Figure 2:**
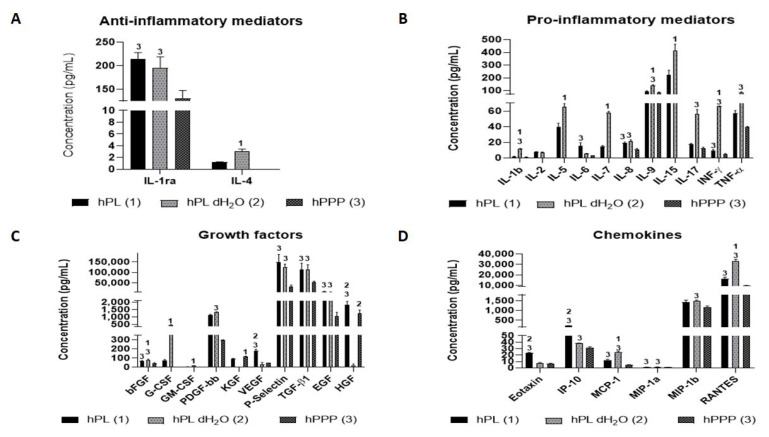
Overview of measured bioactive substances in individual components of platelet lysate. (**A**) Anti-inflammatory mediators; (**B**) pro-inflammatory mediators; (**C**) growth factors; (**D**) chemokines.Statistical analysis *p* < 0.05. The level of significance is denoted by the numbers above the bars in the graph. Tested groups: human platelet lysate—hPL group (1); human platelet lysate in deionized water—hPL dH_2_O group (2); platelet poor plasma—hPPP group (3). The significance was only denoted with a number above the bars with the significantly higher values.

Selected proteins were analyzed by BioPlex and enzyme-linked immunosorbent assay (ELISA) methods (Figure 2). It was observed that the concentrations of IL-1B, IL-4, IL-5, IL-7, IL-9, IL-15, INF-γ, bFGF, GM-CSF, G-CSF, VEGF, MCP-1 and RANTES were significantly higher in the group hPL dH_2_O in comparison to the group hPL. The concentrations of IL-2, IL-4, IL-5, IL-7, IL-15, G-CSF, GM-CSF were under the detection limit in the group hPPP. The highest values were recorded for the chemotactic factor RANTES in all groups. KGF concentrations were significantly higher in the plasma (hPPP) than in the platelet lysate (hPL). Moreover, the concentration of KGF in platelets (hPL dH_2_O) was under the detection limit.

VEGF concentration was significantly higher in the platelet lysate (hPL), compared to the platelets (hPL dH_2_O) and plasma (hPPP).

Concentration of P-selectin as a marker of platelet activation was significantly higher in the samples containing platelets (hPL, hPL dH_2_O), compared to platelet poor plasma (hPPP). The same results were observed for TGF-β and EGF concentrations. On the other hand, HGF concentration was significantly higher in the samples with plasma (hPL, hPPP), compared to the separated platelets (hPL dH_2_O).

### 2.2. The Effect of Individual Platelet Lysate Components on 3T3 Fibroblasts

**Figure 3:**
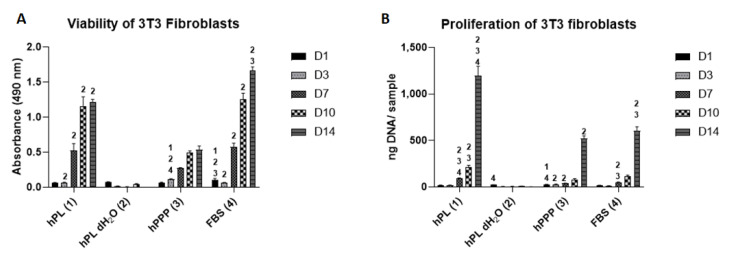
Viability and proliferation of 3T3 fibroblasts supplemented with individual platelet lysate components. (**A**) Viability of 3T3 fibroblasts measured by MTS assay. (**B**) Proliferation of 3T3 fibroblasts. Statistical analysis *p* < 0.05. The level of significance is denoted by the numbers above the bars in the graph. The numbers above the bars denote the type of the respective sample 1—hPL, 2—hPL dH_2_O, 3—hPPP. The significance was only denoted with a number above the bars with the significantly higher values. Number of experiments: *n* = 6.

Cell metabolic activity was detected using MTS assay. Cells had better adherence to the surface when supplemented with FBS (day 1 of experiment, Figure 3A). The viability steadily grew in the groups supplemented with plasma and FBS. On day 3, the absorbance was significantly the highest in hPPP, and higher in hPL and FBS, compared to the group supplemented with hPL dH_2_O. The cell metabolic activity in the group supplemented with hPL dH_2_O gradually decreased throughout the experiment. From day 7 until day 14, hPL and FBS groups showed significantly higher absorbance compared to hPL dH_2_O.

The fibroblast proliferation was evaluated by a fluorescence-based assay on days 1, 3, 7, 10 and 14 of the experiment (Figure 3B). All of the tested groups, except for the group supplemented with hPL dH_2_O, showed a gradual increase of proliferation. The proliferation was significantly higher in the group supplemented with hPL, compared to all the other groups on days 7 and 14. All of the groups showed significantly higher proliferation, compared to the group supplemented with hPL dH_2_O on day 14. FBS showed higher proliferation, compared to both dPL dH_2_O and hPPP on days 7 and 14. The results indicate the insufficient effect of separate platelets for supporting proliferation, which stagnated during the whole experiment.

**Figure 4:**
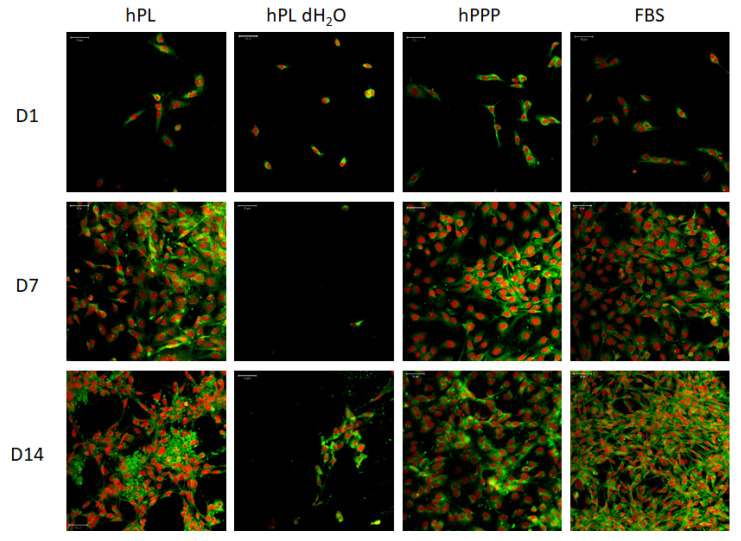
Confocal images of 3T3 fibroblasts supplemented with individual platelet lysate components. Cellular membranes were stained by DiOC6(3) (green color), cell nuclei using propidium iodide (red color). For each sample, a representative image from days 1, 7 and day 14 is given. Objective 20×, scale bar 50 µm. Number of experiments: *n* = 6.

The confocal microscopy images (Figure 4) showed well-spread cells in all groups, except for the group supplemented with hPL dH_2_O. The morphology was typical for 3T3 fibroblasts. In the group supplemented with hPL dH_2_O, the cells created clusters suggesting unfavorable culture conditions. The results from the confocal microscopy were in concordance with the results obtained in the viability and proliferation assays (Figure 3).

### 2.3. The Effect of Individual Platelet Lysate Components on MSCs

**Figure 5:**
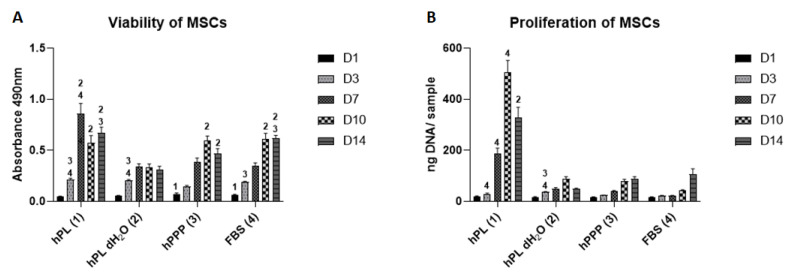
Viability and proliferation of MSCs supplemented with individual platelet lysate components. (**A**) Viability of MSCs measured by MTS assay. (**B**) Proliferation of MSCs. Statistical analysis *p* < 0.05, the level of significance is denoted by the numbers above the bars in the graph. The numbers above the bars denote the type of the respective sample 1—hPL, 2—hPL dH_2_O, 3—hPPP. The significance was only denoted with a number above the bars with the significantly higher values. Number of experiments: *n* = 6.

The viability of MSCs was measured by MTS assay (Figure 5A). The initial adhesion of cells was significantly improved in the group supplemented with plasma (hPPP) and FBS, compared to the group supplemented with platelet lysate (hPL) on day 1 of the experiment. Throughout the experiment, the metabolic activity steadily increased in the group supplemented with FBS. The group with the platelet lysate (hPL) showed an increase in metabolic activity between days 3 and 7; the metabolic activity then slowly decreased. Despite the observed decrease, the metabolic activity was significantly higher when compared to the hPL dH_2_O. The viability of cells in the group with platelets (hPL dH_2_O) slowly increased until day 7 and then stagnated. The group supplemented with plasma (hPPP) and the control group (FBS), showed significantly higher viability from day 10, compared to the group hPL dH_2_O.

The cell proliferation gradually increased in all the tested groups (Figure 5B), except for the group with platelet lysate (hPL). In the hPL group, an increase of cell proliferation was detected between days 7 and 10; subsequently the slight decrease of both metabolic activity and proliferation were observed, which is typical for confluent cell layers. Proliferation in the group supplemented with platelet lysate (hPL) was significantly higher, when compared to the control group (FBS) on days 7 and 10; moreover, on day 14, it was significantly higher when compared to the group supplemented with platelets (hPL dH_2_O). The results are in concordance with the MTS assay.

**Figure 6:**
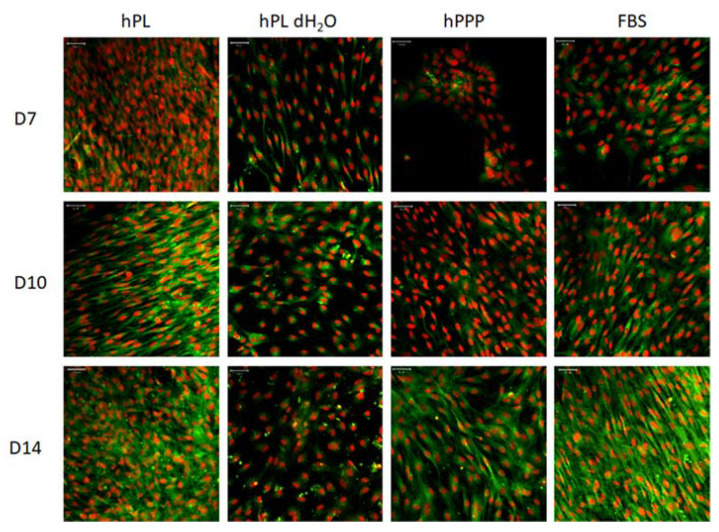
Confocal images of MSCs supplemented with individual platelet lysate components. Cellular membranes were stained by DiOC6(3) (green color), cell nuclei using propidium iodide (red color). For each sample, a representative image from days 7, 10 and day 14 is given. Objective 20×, scale bar 50 µm. Number of experiments: *n* = 6.

Confocal microscopy images (Figure 6) showed that cells proliferated well in all the tested groups, with the exception of the platelet-supplemented group (hPL dH_2_O). In the hPL dH_2_O group the cells were well spread, but the number of cells was significantly lower than in the other groups. The cells in the group supplemented with platelet lysate (hPL) were almost confluent as early as day 7 of the experiment.

## 3. Discussion

During the preparation of platelet lysate there is a need to decrease the plasma concentration in platelet products, due to the interference of plasma proteins with the immune system in the case of allogeneic administration. However, proteins present in the plasma may positively influence the wound healing process; therefore, decreasing the plasma concentration could possibly decrease the efficiency of the healing, mainly in the autologous administration. Therefore, we move towards the verification of concentrations of bioactive molecules contained in the platelet lysate with plasma (hPL), platelet lysate in deionized water (hPL dH_2_O), and platelet poor plasma (hPPP). Moreover, the effect of the bioactive molecules on the proliferation and viability of fibroblasts and MSCs was observed. The group supplemented with FBS served as a control.

Results from the multiplex assay showed significantly higher concentrations of IL-1B, IL-4, IL-7, IL-9 IL-15, IFN-γ, bFGF, G-CSF, GM-CSF, MCP-1 a RANTES in the hPL dH2O group, in comparison with the groups hPL and hPPP. The chosen bioactive molecules were also quantified by the ELISA method. The incubational condition optimized for the binding of the particular protein is the biggest advantage of this method in comparison to the multiplex assay. A higher concentration of TGF-β, P-selectin and EGF was found in the formulations with platelets, in comparison to the hPPP. The preservation of platelet proteins in dH_2_O (hPL dH_2_O) resulted in an insignificantly lower release of KGF, VEGF and HGF, in comparison to the groups with plasma (hPL, hPPP). Surprisingly, in the case of KGF and partially HGF, we observed their high accumulation in platelet poor plasma (hPPP).

The differences between the concentrations of bioactive molecules in hPL and hPPP can be related to the theory of existence of numerous fractions of α-granules. Generally, there are two contradictory theories. The first of these assumes the random distribution of bioactive molecules between the granules, not in the specific subpopulation [31,32]. The second theory assumes the allocation of α-granules to a particular subpopulation with specific bioactive molecules. This theory is supported by studies in which the different effects of various agonists on the release of bioactive molecules were observed [33,34,35,36]. The obtained results suggest that KGF and HGF are secreted from platelets during their storage; therefore, KGF and HGF are accumulated in plasma. Their partial secretion is further supported by the partial release of soluble P-selectin (a marker of α-granule release in the hPPP group). The released amount was lower in the hPPP group than in the samples containing platelets postlysis, which indicates that a significant number of α-granules were not released before the lysis.

The differences in the release between the samples containing plasma (hPL, hPPP) and deionized water (hPL dH_2_O), might be connected to binding to the carrier proteins such as fibrinogen, albumin and the other components of blood plasma. Since the protein concentration in the hPL dH_2_O group was significantly lower, the sequestration was rather limited; no protein binding during the immunodetection methods was observed, along with a decrease in the molecule function. Cannon et al. determined IL-1B in human blood plasma and serum. They demonstrated that IL-1B could be bound to the larger carrier proteins, thus hindering its detection [37]. In our study, a lower concentration of IL-1B was detected in the plasma-contained samples than in the hPL dH_2_O group. However, protein sequestration can be important in binding the receptor and coreceptor in target cells. Sahni et al. observed the binding of IL-1B to fibrinogen. The results showed that IL-1B, in comparison to IL-1A, binds with a high affinity to the fibrinogen, and hence its activity is increased [27]. The binding to carrier proteins is important for the signal molecules. Martino et al. observed the binding of growth factors to fibrinogen through a heparin-binding domain. They confirmed that growth factors from the PDGF/VEGF, FGF and TGF-B family, as well as growth factors from the neurotrofin family, were bound to fibrinogen. Growth factors, which were not bound to fibrinogen, were rapidly released from a fibrin matrix, while the bound growth factor was retained. PDGF-BB showed a strong binding to fibrinogen; however, it was rapidly released from the matrix [38]. In our experiment, the concentration of PDGF-BB was shown to be similar in the hPL and hPL dH_2_O samples. It should be noted that the samples with the heparinised plasma may contain precipitated proteins after repeated freeze/thawing and, therefore, their identification may be difficult [39,40]. Storing samples with cellular components for more than two hours also significantly alters the concentration of some cytokines that are either degraded, absorbed, or produced by cells [41]. The above-mentioned effects may also affect other types of mediators for which they have not yet been assessed.

Fibroblasts are the key cells of connective tissue formed by the differentiation of cells of mesenchymal origin, with particular importance in the regeneration of connective tissues, skin and internal wounds. Fibroblasts adhered similarly in all groups when different supplements were used. However, throughout the experiment their metabolic activity and proliferation stagnated in the hPL dH_2_O group. One reason may be TNF-α, as its level was 1.5 times higher in the platelet-only group (hPL dH_2_O) than in both the plasma and the platelet-containing group (hPL and hPPP). It is a pleiotropic cytokine that has various functions, including the induction of apoptosis in many cell types, including fibroblasts [42]. Frankel et al. studied the effect of TNF-α on normal and fibrotic lung fibroblasts. They found that the basal resistance of fibroblasts to Fas-induced apoptosis was overcome by sensitization TNF-α [43]. Graves et al. performed an experiment in which they inoculated wild-type mice and mice without the TNF receptor (TNFR) with porphyromonas gingivalis. Inoculation with the bacteria stimulated TNF production and fibroblast apoptosis, but was significantly reduced in TNFR −/− mice. This suggests that the bacteria stimulates TNF production, which causes the programmed cell death of fibroblasts [44]. Another reason may be that the higher concentration of IFN-γ. IFN-γ affects a number of cellular functions, such as modulation of the immune response and the induction of cell differentiation, but also an inhibition of cell growth [45,46]. Most frequently, IFN-γ blocks the cell cycle in the G1/S phase. IFN-γ transcriptionally induces p21 and p27 inhibitors of cyclin-dependent kinases that inhibit the activity of cyclin E:CDK2 and cyclin D:CDK4 complexes, thereby blocking the cell cycle at the G1/S [47]. Significantly higher concentrations were measured in the hPL dH_2_O sample, compared to the other samples. This difference may have a significant inhibitory effect on fibroblast proliferation and viability in the hPL dH_2_O sample. In a study by Wang et al., the apoptosis of mitomycin-resistant fibroblasts was monitored. The combination of IFN-γ has been found to make these fibroblasts sensitive to Fas-mediated apoptosis. INF-γ and INF-α have a synergistic effect on sensitization [48]. Another reason could be the absence of stimulatory factors. This is the absence of plasma proteins, namely fibrinogen and albumin. Albumin is one of the most abundant plasma proteins. A study by Todaro et al. showed a positive effect on fibroblast proliferation by adding serum albumin to the culture medium [49]. The albumin level was below the detection limit in the plasma-free sample (hPL dH_2_O), which could also affect fibroblast viability. Moreover, in the hPL dH_2_O group, the concentration of numerous growth factors was lower. These factors included IGF-I, a low amount of which was detected in the hPL dH_2_O. IGF-I is an important trophic factor synthesized in the liver; it circulates in plasma but can also be found in α-granules of platelets [50]. IGF-I stimulates the DNA synthesis of fibroblasts [51] and leads to an increase in extracellular matrix synthesis, namely collagens and proteoglycans [52]. In this experiment, the concentration of IGF-I was almost identical in the samples containing plasma (hPL, hPPP) while in the plasma-free sample (hPL dH_2_O), IGF-I concentration was below the detection limit. The concentration of IGF-I may affect the fibroblasts’ behavior. Furthermore, lower concentrations of EGF, HGF and KGF were measured in the plasma-free sample (hPL dH_2_O). However, cytokines such as PDGF, bFGF, TGF-β and EGF were measured at similar concentrations as in the samples containing plasma (hPL, hPPP).

Thus, the inhibitory effect was mainly due to a combination of the above-mentioned effects. The effect of the albumin absence, lack of trophic IGF-I, and high levels of TNF-α and INF-γ appeared to be key for actual low fibroblast proliferation/viability in a hPL dH_2_O group.

Proliferation and metabolic activity were also lower in the group supplemented with platelet-poor plasma (hPPP). Low levels of inhibitory cytokines, such as TNF-α, INF-γ and IL-1B, were observed in this group. Plasma protein concentrations were comparable to the hPL group. However, the concentrations of stimulatory growth factors, such as bFGF, VEGF, PDGF-bb, G-SCF, GM-SCF, TGF-β and EGF, were lower. Thus, the lower rate of proliferation and metabolic activity may be attributed to the lower concentration of these factors.

Overall, the obtained results stress the importance of plasma proteins and IGF-I, as their absence inhibits the fibroblast growth. A combination with growth factors contained in platelets further stimulates the fibroblast growth, and this synergistic effect is advantageous. The best supplement seemed to be hPL, in which fibroblasts achieved statistically similar results in metabolic activity testing and better results in proliferative capacity testing, when compared to the FBS supplemented group. Thus, hPL appears to be a suitable alternative to FBS. Plasma elimination and its substitution with dH_2_O, is not possible without further optimization. Appropriate levels of albumin, fibrinogen and IGF-I, seem to play a key role in cell stimulation. However, due to the high concentrations of inhibitory cytokines (TNF-α, INF-γ), which may reduce the stimulatory effects, successful formulation still needs further investigation.

Furthermore, the tested groups were used as culture medium supplements for MSCs. On day one of the experiment, the observed adhesion of the MSCs was similar in all tested groups. However, their viability and proliferation varied during the experiment. The group with the plasma-free supplement (hPL dH_2_O) did not provide adequate nutrition to the cells, causing them to proliferate very slowly. However, the trend of a gradual increase in DNA content and viability was also visible in this group. A very pronounced stimulatory effect on cell proliferation was achieved in the hPL group, where the effect was significantly stronger than in other groups (including the FBS group).

The effects of different cytokines (such as inhibition by TNF-α [53] and INF-γ [54]) were similar in a culture of MSCs. Wang et al. have shown the damage of MSC self-renewal and differentiation, by the NFκB signaling cascade [54]. Thus, the incubation of MSCs with these bioactive molecules results in inhibition, as observed in the cultured fibroblasts. However, the effect of IL-1B and IL-6 on MSCs is different from the effect observed in fibroblasts. These interleukins stimulate their stemness and immunomodulatory functions [55,56,57], and their activity increases the MSC’s paracrine signaling and leads to cell proliferation. In contrast, IGF-I deficiency is critical for MSCs. IGF-I signaling has a proliferative effect, an antiapoptotic effect, and is important for appropriate bFGF signaling [58]. Thus, in the dH_2_O group (hPL dH_2_O), there is an absence of IGF-I signaling, thereby reducing the proliferation in MSCs. Consequently, the cumulative effect of the absence of IGF-I, and high concentrations of TNF-α and INF-γ, is likely to cause low metabolic activity and proliferation of MSCs in the hPL dH_2_O group.

Similarly to fibroblasts, the higher proliferation of MSCs in the hPL group was due to the presence of proliferative growth factors, such as IGF-I [58], bFGF [59], TGF-β [60], PDGF-bb, EGF [61], G-SCF [62] and GM-SCF [63]. The positive effect of these factors was also evident in comparison with the FBS group. FBS is the standard growth supplement, but may cause the risk of immunogenicity in human mesenchymal stem cell (hMSC) cultures [64,65]. FBS is also affected by its insufficient characterization and large qualitative and quantitative variability of components (seasonal and geographical variability between batches) [66]. In MSCs, platelet-derived factors increased proliferation. The results were consistent with the studies by Mishra et al. [67] and Vogel et al. [68], where PRP improved MSC expansion.

## 4. Materials and Methods

### 4.1. Platelet Lysate Preparation

Fresh human platelet concentrate derived from buffy coat, was purchased from the blood transfusion unit (ÚHKT, Prague, Czech Republic). Platelet concentrate was prepared from the blood of 16 donors to minimize interindividual variability. The platelet concentrate was divided into three parts. The first part was utilized for platelet lysate preparation (human platelet lysate (hPL group)). The second part was centrifuged (3389× *g*/10 min; break set to off). Subsequently, the supernatant was replaced with deionized water and this platelet concentrate was also lysed (human platelet lysate deionized water (hPL dH_2_O group)). The last part was centrifuged (3389× *g*/10 min; break set to off) and the supernatant was used for the experiment (human platelet poor plasma (hPPP group)) (Table 2). The groups with platelets were lysed by the freeze (−80 °C)/thawing (37 °C) method (three times), and centrifuged (3389× *g*/10 min; break set to off)) for removing the cellular membranes. All groups were stored at −80 °C until use. The study was conducted in accordance with the Institute of Experimental Medicine CAS, and the protocol was approved 28 April 2020 by the Ethics Committee of the Institute of Experimental Medicine CAS under the file No 2020/04.

### 4.2. Cell Culture and Seeding

A murine 3T3-A21 cell line (fibroblasts) was purchased from Sigma-Aldrich, and primary human mesenchymal stem cells derived from bone marrow (MSC) were purchased from ScienCell (Carlsbad, CA, USA). The 3T3 fibroblasts were cultured in DMEM supplemented with 10% fetal bovine serum (FBS; Sigma-Aldrich, St. Louis, MO, USA), and treated with penicillin/streptomycin (100 IU/mL, 100 μg/mL). The mesenchymal stem cells were cultured in alpha MEM supplemented with 10% FBS, and treated with penicillin/streptomycin (100 IU/mL, 100 μg/mL). All media types were refreshed every 3–4 days.

The cells were seeded at a density of 10,000 cells/cm^2^, for both fibroblasts and MSCs. The cells were cultured in the presence of 8% (*v*/*v*) platelet lysate components. A medium with 10% FBS served as a control. Media containing platelet components were treated with heparin (2 IU/mL), to prevent fibrin clot formation.

### 4.3. Quantification of the Overall Protein in Platelet Lysate Components

The overall protein contained in platelet lysate components was quantified using a fluorescence kit (Quant-iT-Protein Assay Kit, Invitrogen Life Technologies, Carlsbad, CA, USA). From each sample 10 µL was taken and 200 µL of protein kit working solution was added. Samples were placed in a black 96-well plate (Corning 3603, New York, NY, USA) and the fluorescence of individual samples was measured using a Tecan reader (Tecan Group Ltd., Männedorf, Switzerland) at excitation values of 470 nm, emission 570 nm. To compile the calibration curve, standards were plotted in doublets (part of the kit, protein content 0–500 ng/µL) and the concentration of total protein in individual samples was calculated on the basis of the calibration curve.

### 4.4. Quantification of Fibrinogen and Albumin in Platelet Lysate

To specify the concentration of fibrinogen and albumin in the platelet lysate, the samples were characterized in the diagnostic laboratory Synlab Czech (Prague, Czech Republic) according to the manufacturer’s protocol. To measure the concentration of albumin, bromocresol green dye was used. The absorbance of bromocresol green/albumin complex was detected using AU5800 reader (Beckman Coulter, Brea, CA, USA). The mass concentration of fibrinogen was determined by modified Claus method using an automatic coagulometer Sysmex CA 1500 (Siemens, Munich, Germany). 

### 4.5. Quantification of IGF-I

Quantification of IGF-1 was performed using the immunoradiometric method in the laboratory of Imalab (Zlin, Czech Republic). Mouse monoclonal antibodies directed against two different noncompetitive epitopes of IGF-I were used. A previous dissociation step is required to release IGF-I from protein binding. Samples and calibrators were incubated in tubes coated with the first monoclonal antibody in the presence of a second iodine-125 labeled monoclonal antibody. After incubation, the contents of the tubes were removed and bound radioactivity was measured. Unknown values were determined by interpolation from a standard curve. Bound radioactivity is directly proportional to the concentration of IGF-I in the sample.

### 4.6. Quantification of Selected Growth Factors in Platelet Lysate

The concentrations of growth factors (HGF, EGF, VEGF, P-selectin, KGF, TGF-β) in the platelet lysate and its individual components were determined by ELISA (Enzyme-Linked ImmunoSorbent Assay) using the Duo Set antibody system (R&D Systems, Minneapolis, MN, USA). The surface of the 96-well plate was first incubated with the primary antibody for 12 h at room temperature and then blocked with phosphate buffer (PBS) with 1% bovine serum albumin (BSA). At room temperature, the primary antibody was incubated for 2 h with samples and standards. Detection was performed by incubation with biotinylated primary antibody (2 h, room temperature) and streptavidin-HRP (avidin-peroxidase) conjugate (20 min, room temperature). Between steps, the wells in the plate were washed with PBS with 0.05% Tween-20. HRP-bound activity was determined colorimetrically by adding TMB (3,3′,5,5′-Tetramethylbenzidine, Life Technologies, Carlsbad, CA, USA) substrate and measuring absorbance at 450 nm on a Biotek Synergy H1 ELISA reader spectrophotometer (Biotek, Winooski, VT, USA) after 20 min of reaction at room temperature. The concentrations of growth factors were determined on the basis of calibration curves.

### 4.7. Quantification of Cytokines in Platelet Lysate

The BioPlex system (Bio-Rad Laboratories, Hercules, CA, USA) was used to detect selected bioactive substances contained in hPL. This method makes it possible to determine a large number of analytes in one sample at a time. Samples were diluted 1:4 in diluent. The standard was reconstituted and diluted 4-fold. Antibody-coated magnetic beads were prepared and placed in an assay plate. The magnetic bead solution was vortexed thoroughly before each addition to the plate. The plate was washed on an automatic washer. After washing, standards and samples were added to the wells of the beads. The plate was incubated on a shaker and, after incubation and washing, the detection antibody was added. The plate was incubated again on a shaker and streptavidin-phycoerythrin was added after the next washing step. After the last incubation step, the plate was placed in the instrument and analyzed. The results were analyzed using Bio-Plex Manager software. Absolute sample concentrations were evaluated by constructing a calibration curve for the analyte. The following bioactive substances were evaluated: interleukin-1b (IL-1b), interleukin-1ra (IL-1ra), interleukin-2 (IL-2), interleukin-4 (IL-4), interleukin-5 (IL-5), interleukin-6 (IL-6), interleukin-7 (IL-7), interleukin-8 (IL-8), interleukin-9 (IL-9), interleukin-10 (IL-10), interleukin-12 (IL-12), interleukin-13 (IL-13), interleukin-15 (IL-15), interleukin-17 (IL-17), granulocyte colony stimulating factor (G-CSF), granulocyte-macrophage colony stimulating factor (GM-CSF), interferon-gamma (INF-γ), tumor necrosis factor-α (TNF-α), MCP-1 (Monocyte Chemoattractant Protein-1), CXCL10 chemokine (IP-10), MIP-1a, MIP 1b (CCL-4), RANTES, eotaxin (CCL-11), platelet-derived growth factor (PDGF), basic fibroblast growth factor (bFGF), and vascular endothelial growth factor (VEGF).

### 4.8. Cell Metabolic Activity

The MTS assay (CellTiter96 Aqueous One Solution Cell Proliferation Assay, Promega, Madison, WI, USA) is a colorimetric method that allows monitoring of changes in the metabolic activity of seeded cells. Briefly, to each well with cells 20 µL of reagent solution, (3-3-(4,5-dimethylthiazol-2-yl)-5-(3-carboxymethoxyphenyl)-2-(4-sulfophenyl)-2H-tetrazolium and 100 µL of culture medium was added. The cells were cultured in an incubator for 2 h. A sample of 100 µL of the final color product was moved into a new plate and the absorbance was measured on a spectrophotometer (Infinite M200 PRO; Tecan, Männedorf, Switzerland) at 490 nm and 690 nm for background readings. The absorbance values of the samples were calculated by subtracting the value of the cell-free medium used.

### 4.9. Cell Proliferation

DNA content was determined using a sensitive fluorescent dye (QuantiT-High Sensitivity dsDNA Assay Kit, Invitrogen, Carlsbad, CA, USA). This method is based on the binding of an ultrasensitive fluorescent dye to double-stranded DNA. Cell suspensions were transferred to microtubes with 200 µL of lysis buffer (10 mM Tris, 1 mM EDTA, 0.2% *v/v* Triton X-100). Samples were frozen 3 times (−20 °C) and thawed (room temperature) and vortexed thoroughly between each step. From each sample, 10 µL of solution was placed into a black 96-well plate; which was completed to 200 µL with the kit working solution. The fluorescence of individual samples was measured using a spectrophotometer (Infinite M200 PRO; Tecan, Männedorf, Switzerland) at excitation values of 485 nm and emission of 528 nm. Standards (part of the kit, DNA content 0–10 ng/µL) were used to construct the calibration curve, and the DNA content in the individual samples was calculated on the basis of the calibration curve.

### 4.10. Cell Visualization via Confocal Microscopy

The cells were fixed with methanol (−20 °C), washed with PBS and stained with DiOC6(3) (Cat. No. 318426, Sigma-Aldrich, St. Louis, MO, USA), 1 μg/mL in PBS; 30 min at RT; green color) to visualize the cell membranes and propidium iodide (Cat. No. P4170, Sigma-Aldrich, St. Louis, MO, USA, 5 μL/mL in PBS; 10 min at RT; red color) to visualize the cell nuclei. The samples were scanned using LSM 510 DUO confocal microscope (Zeiss, Oberkochen, Germany) at λex = 488 nm, λem = 505–555 nm for DiOC6(3), λex = 560 nm, λem > 575 nm for propidium iodide, Objective × 20.

### 4.11. Statistical Analysis

Data were evaluated using SigmaStat 3.5 software (Systat, San Jose, CA, USA). Firstly, Kolmogorov−Smirnov was performed to determine the normality of the data. Statistically significant differences between the groups with normal distribution were evaluated by ANOVA, and Tukey’s test was used for post hoc analysis. Data with an abnormal distribution were tested by the Kruskal−Wallis test and the Dunn’s method for post hoc analysis. Statistical significance was accepted at the 5% level (p:0.05). N value was 6 for the cell culture testing and 4 for the bioactive molecule analysis.

### 4.12. List of Abbreviations

3T3-A31 (Murine fibroblast cell line), ADP (Adenosine diphosphate), ANOVA (Analysis of variance), bFGF (Basic fibroblast growth factor), DiOC6(3) (3,3′-dihexyloxacarbocyanine iodide), DMEM (Dulbecco’s Modified Eagle’s Medium), EDTA (Ethylenediaminetetraacetic acid), EGF (Epidermal growth factor), ELISA (Enzyme-linked immunosorbent assay), FBS (Fetal bovine serum), FGF (Fibroblast growth factor), G-CSF (Granulocyte-colony stimulating factor), GM-CSF (Granulocyte-macrophage colony-stimulating factor), HGF (Hepatocyte growth factor), IGF-1 (Insulin-like growth factor-1), IL-1b (Interleukin-1b), IL-1ra (Interleukin-1ra), IL-1α (Interleukin-1α) IL-2 (Interleukin-2), IL-4 (Interleukin-4), IL-5 (Interleukin-5), IL-6 (Interleukin-6), IL-7 (Interleukin-7), IL-8 (Interleukin-8), IL-9 (Interleukin-9), IL-15 (Interleukin-15), IL-17 (Interleukin-17), INF-α (Interferon-α), INF-γ (Interferon-γ), IP-10 (Interferon gamma-induced protein-10), KGF (Keratinocyte growth factor-1), MCP-1 (Monocyte chemoattractant protein-1), MEM (Minimum essential medium), MIP-1a (Macrophage-inflammatory protein-1a), MIP-1b (Macrophage-inflammatory protein-1b), MSC (Human mesenchymal stem cells), MTS (3-(4,5-dimethylthiazol-2-yl)-5-(3-carboxymethoxyphenyl)-2-(4-sulfophenyl)-2H-tetrazolium), p21 (cyclin-dependent kinase inhibitor 1), PAR-1 (Protease-Activated Receptor 1), PAR-4 (Protease-Activated Receptor 4), PBS (Phosphate buffer saline), PDGF-bb (Platelet-derived growth factor bb), PF-4 (Platelet factor 4), RANTES (Regulated on Activation, Normal T Cell Expressed and Secreted), TGF-β (Transforming growth factor-β),TNF-α (Tumor necrosis factor-α), TNFR (Tumor necrosis factor receptor), VEGF (Vascular endothelial growth factor).

## 5. Conclusions

Platelet concentrate and its derivatives are widely used for in vitro cell culture, but also in regenerative medicine to improve the wound healing process [69]. However, the individual components of the platelet concentrate contain different types of bioactive substances, which can have both positive and negative effects on the cellular fate. Plasma, one of the components of platelet concentrate, can cause immunological responses to plasma components when allogeneic products are administered, and it is therefore desirable to replace it for certain applications. In this experiment, the individual components of the platelet concentrate were prepared and characterized for the concentrations of selected bioactive substances and, at the same time, these components were added to the medium and their effect on cell cultures was monitored in vitro. Neither platelet lysate in deionized water nor plasma alone was found to provide sufficient cell proliferation and viability. This is only ensured by the synergistic action of both of these components. Therefore, the effect of the plasma proteins and growth factors, namely albumin and IGF-I, are important for accelerated wound healing.

## Figures and Tables

**Table 1:** Concentrations of fibrinogen, albumin, and IGF-1 in the tested groups.

Sample	Fibrinogen Concentration (g/L)	Albumin Concentration (g/L)	IGF-I Concentration (ng/mL)
hPL	0.50	8.47	32.43
hPL dH_2_O	<0.50	<2.00	<11.15
hPPP	0.64	8.31	33.79

**Table 2:** Summary of individual components of platelet concentrate.

Sample Type	Abbreviation
Human platelet lysate	hPL
Human platelet lysate in deionized water	hPL dH_2_O
Human platelet poor plasma	hPPP

## Data Availability

The data that support the findings of this study are available from the corresponding author upon reasonable request.
